# Multiple roles of GluN2B-containing NMDA receptors in synaptic plasticity in juvenile hippocampus

**DOI:** 10.1016/j.neuropharm.2016.08.010

**Published:** 2017-01

**Authors:** Grace France, Diego Fernández-Fernández, Erica S. Burnell, Mark W. Irvine, Daniel T. Monaghan, David E. Jane, Zuner A. Bortolotto, Graham L. Collingridge, Arturas Volianskis

**Affiliations:** a Centre for Synaptic Plasticity, School of Physiology, Pharmacology and Neuroscience, University of Bristol, Bristol, UK; b Centre for Synaptic Plasticity, School of Clinical Sciences, University of Bristol, Bristol, UK; c Department of Pharmacology and Experimental Neuroscience, University of Nebraska Medical Center, Omaha, USA; d Dept Physiology, University of Toronto and Lunenfeld-Tanenbaum Research Institute, Mount Sinai Hospital, Toronto, Canada; e Centre for Neuroscience and Trauma, Blizard Institute, Barts and The London School of Medicine and Dentistry, Queen Mary University of London, UK

**Keywords:** Long-term depression, LTD, Short-term potentiation, STP, Long-term potentiation, LTP, NMDA receptors

## Abstract

In the CA1 area of the hippocampus *N*-methyl-d-aspartate receptors (NMDARs) mediate the induction of long-term depression (LTD), short-term potentiation (STP) and long-term potentiation (LTP). All of these forms of synaptic plasticity can be readily studied in juvenile hippocampal slices but the involvement of particular NMDAR subunits in the induction of these different forms of synaptic plasticity is currently unclear. Here, using NVP-AAM077, Ro 25-6981 and UBP145 to target GluN2A-, 2B- and 2D-containing NMDARs respectively, we show that GluN2B-containing NMDARs (GluN2B) are involved in the induction of LTD, STP and LTP in slices prepared from P14 rat hippocampus. A concentration of Ro (1 μM) that selectively blocks GluN2B-containing diheteromers is able to block LTD. It also inhibits a component of STP without affecting LTP. A higher concentration of Ro (10 μM), that also inhibits GluN2A/B triheteromers, blocks LTP. UBP145 selectively inhibits the Ro-sensitive component of STP whereas NVP inhibits LTP. These data are consistent with a role of GluN2B diheretomers in LTD, a role of both GluN2B- and GluN2D- containing NMDARs in STP and a role of GluN2A/B triheteromers in LTP.

This article is part of the Special Issue entitled ‘Ionotropic glutamate receptors’.

## Highlights

• Inhibition of GluN2Bs in P14 is sufficient for blockade of NMDAR-LTD.
• GluN2A and GluN2D subunits are not required for the induction of LTD.
• Induction of STP involves GluN2B and GluN2D subunits.
• Induction of LTP depends on GluN2A/2B triheteromers.

## 1 Introduction

*N*-Methyl-d-aspartate receptors (NMDARs) are centrally involved in synaptic transmission, synaptic plasticity and learning and memory (Bliss and Collingridge, 2013, Bliss and Collingridge, 1993, Volianskis et al., 2015). NMDARs are glutamate gated ion channels that form heterotetrameric complexes. They usually consist of two GluN1 subunits and two GluN2 subunits, of which there are four possible types (GluN2A, 2B, 2C, 2D) (Collingridge et al., 2009). At the majority of excitatory synapses, and notably the CA1 area of the hippocampus, the induction of long-term potentiation (LTP) and one form of long-term depression (LTD) requires NMDA receptor activation. Use of the NMDAR specific antagonist, D-AP5, demonstrated the receptor involvement in LTP (Collingridge et al., 1983) and *de novo* LTD (Dudek and Bear, 1992) in rodent hippocampal slices.

The role of NMDAR's in synaptic plasticity has been studied extensively (Collingridge et al., 1983, Tsien et al., 1996); however, there has been considerable disagreement about the involvement of specific receptor subtypes in both LTP and LTD (Bartlett et al., 2007, Berberich et al., 2005, Cull-Candy et al., 2001, Hrabetova et al., 2000, Li et al., 2007, Liu et al., 2004, Massey et al., 2004, Paoletti et al., 2013, Sakimura et al., 1995, Tang et al., 1999). One suggestion that has gained considerable traction is that GluN2A-containing NMDARs are required for LTP and that GluN2B-containing NMDARs are involved in LTD (Bartlett et al., 2007, Köhr et al., 2003, Liu et al., 2004, Massey et al., 2004, Sakimura et al., 1995). However, there is also considerable evidence that GluN2B-containing NMDARs are important for LTP (Barria and Malinow, 2005, Bartlett et al., 2007, Berberich et al., 2007, Tang et al., 1999) and the role of GluN2B-containing NMDARs in LTD has been challenged (Bartlett et al., 2007, Li et al., 2007, Morishita et al., 2007).

It is possible that there are age-dependent differences in experimental observations due to a developmental change in subunit composition of NMDARs with GluN2As being expressed less at younger ages (Barria and Malinow, 2005, Loftis and Janowsky, 2003, Monyer et al., 1994). This cannot, however, be the only explanation since at a given stage of development there is also controversy regarding the role of GluN2A and GluN2B subunits (Berberich et al., 2007, Berberich et al., 2005, Liu et al., 2004, Morishita et al., 2007).

Another complicating factor could be in the selectivity profiles of the pharmacological agents that are commonly used to investigate the role of the different NMDA receptor subtypes. The most commonly used antagonists have a narrow selectivity window or, in the case of ifenprodil-like GluN2B antagonists, a complex mode of action (Fischer et al., 1997, Hansen et al., 2014).

Recently we rigorously characterized three antagonists NVP-AAM077 (NVP), Ro 25-6981 (Ro) and UBP145 and showed that they could be used to identify the roles of GluN2A, GluN2B-containing diheteromers, GluN2A/B triheteromers and GluN2D-containing NMDARs in synaptic plasticity in the CA1 region of adult rat hippocampal slices (Volianskis et al., 2013a). We found that the predominant receptor required for the induction of LTP was the GluN2A/B triheteromer. In addition we found that a significant component of short-term potentiation (STP), an initial decremental phase of LTP that is observed following high frequency activation and low frequency test stimulation (Volianskis and Jensen, 2003), involved both GluN2B and GluN2D subunits (Volianskis et al., 2013a).

In the present study we have investigated the role of NMDAR subunits in LTP and *de novo* LTD in P14 animals using these three antagonists. In particular, we also sought to establish the role of GluN2B and GluN2D-containing NMDARs (GluN2B, GluN2D) in rats of this age.

## 2 Materials and methods

### 2.1 Compounds

D-(-)-2-Amino-5-phosphonopentanoic acid (D-AP5) and (α*R*,β*S*)-*α*-(4-hydroxyphenyl)-*β*-methyl-4-(phenylmethyl)-1-piperidinepropanol maleate (Ro 25-6981) were purchased from Tocris Bioscience (Bristol, UK). (*R*)-[(*S*)-1-(4-bromophenyl)ethylamino]-(2,3-dioxo-1,2,3,4-tetrahy-droquinoxalin-5-yl)-methyl]phosphonic acid (NVP-AMM077) and (2R*,3S*)-1-(9-bromophenanthrene-3-carbonyl)piperazine-2,3-dicarboxylic acid (UBP145) were synthesised in house as described previously (Costa et al., 2009, Irvine et al., 2012).

### 2.2 Electrophysiology

Experiments were performed as described previously (Bartlett et al., 2007), following institutional approval and according to the national and EU guidelines for animal care. Briefly, P14 Wistar rats were decapitated following cervical dislocation (UK Scientific Procedures Act, 1986). Hippocampi were dissected and sliced with a Microslicer (DSK DTK-1000). Parasagittal slices (400 μm) were placed in an interface chamber and perfused with aCSF solution containing 124 mM NaCl, 26 mM NaHCO_3_, 3 mM KCl, 1.4 mM NaH_2_PO_4_, 1 mM MgSO_4_, 2 mM CaCl_2_ and 10 mM d-glucose, saturated with 95% O_2_/5% CO_2_ at 28 °C.

Test stimuli (100 μs) were delivered at 0.033 Hz through bipolar nickel-chromium electrodes, which were placed in the CA1 area of the hippocampal slice to stimulate the Schaffer collateral fibres. Field excitatory postsynaptic potentials (fEPSPs) were recorded using glass microelectrodes filled with 3 M NaCl solution (resistance ∼2–5 MΩ) and positioned in the *stratum radiatum* of the CA1. A 30 min baseline was recorded at a stimulus intensity that gave 60–70% of the maximal response. LTD was induced by low frequency stimulation (LFS, 1 Hz stimulation for 15 min) and LTP was induced using high frequency stimulation (HFS, 100 Hz for 1 s). The data were collected and analysed using WinLTP (Anderson and Collingridge, 2007).

Extracellular fEPSP recordings were amplified using an Axoclamp 2B amplifier (Axon Instruments, Foster City, CA), filtered at 1–3 kHz and digitised at 20 kHz (CA-1000, National Instruments). The early slopes of the fEPSPs were measured starting at the point of the fibre volley termination (0.2–0.5 ms). Post-LFS/HFS responses were normalised to baseline.

### 2.3 Data analysis

A single slice from one animal was used for each experimental group (hence n values refer to both the number of slices and the number of animals) and pharmacological experiments were randomized and interleaved with controls. Data are presented as mean values ± SEM. The LTD/LTP levels were estimated at the end of each single experiment (1 h post LFS/HFS) from 4 min averages, generating the mean values for each of the groups. The values from the single experiments were used for the statistical comparison. Significance of LTD/LTP was assessed using paired t-tests when comparing to the pre-LFS/HFS baseline. One-way ANOVAs with Bonferroni post-hoc tests were used to compare the normalised fEPSP slopes between the groups (SigmaPlot). Decay times of STP were analysed as described previously (Volianskis et al., 2013a, Volianskis and Jensen, 2003). Briefly, decay of STP was fitted using a mono-exponential fitting routine in GraphPad Prism and statistical comparisons between decay time constants (τ) were done with extra sum-of-squares F-test (Prism). τ values are presented together with their confidence interval (CI). Statistical differences were set at p < 0.05.

## 3 Results

### 3.1 GluN2A- and GluN2D-preferring antagonists have no effect on LTD

**Fig. 1:**
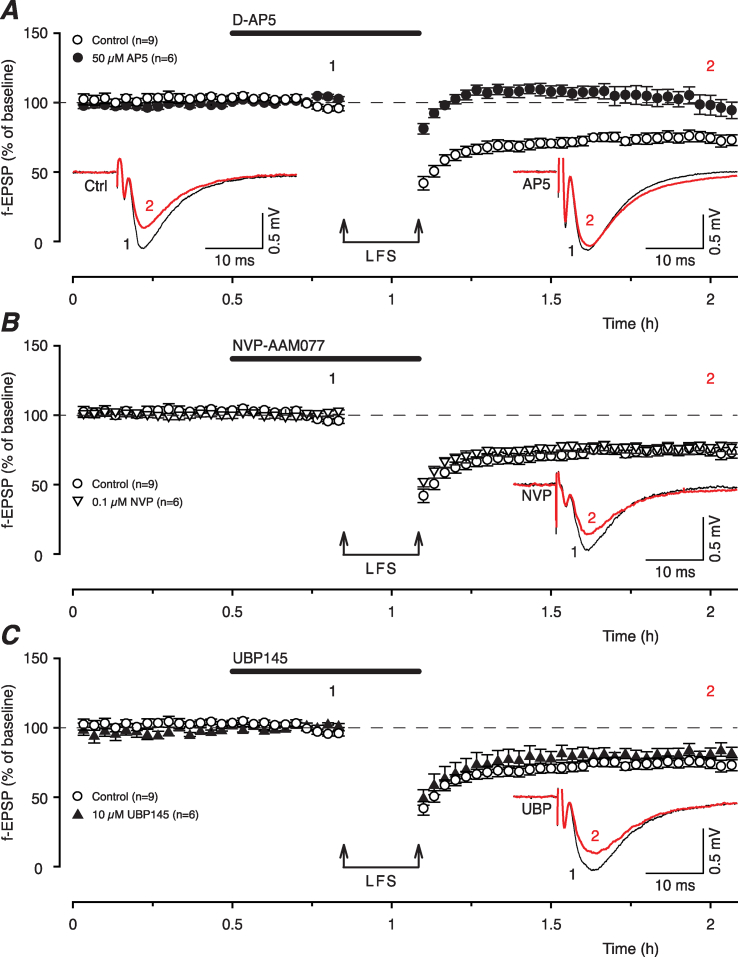
GluN2A and GluN2D subunits are not required for the induction of LTD. (**A**) LTD induced by low frequency stimulation (LFS, 900 stimuli @ 1 Hz; open circles; n = 9) is readily blocked by application of 50 μM D-AP5 (filled circles, n = 6). Insets show representative field responses from a control experiment (Ctrl) and from an AP5 experiment. (**B**) 0.1 μM NVP (open triangles; n = 6) and (**C**) 10 μM UBP145 (UBP, filled triangles; n = 6) are ineffective in blocking LTD. In this and subsequent figures, each point plots the mean ± SEM. The example traces were obtained at the times indicated by colour-coded numbers, the stimulation artefacts have been truncated for demonstration purpose in this and all the subsequent figures. The duration of the application of compounds is indicated by bars. The same set of interleaved control experiments is plotted in each panel for ease of comparison.

Low frequency stimulation (LFS, 1 Hz 900 stimuli) induced robust LTD in P14 hippocampal slices (Fig. 1A, open circles, 26 ± 4% 1 h post-induction; n = 9, p < 0.05 when compared to the pre-LFS baseline, paired *t*-test). A 20-min long pre-application of AP5 (50 μM) prevented LTD demonstrating NMDAR involvement in the induction of synaptic depression (Fig. 1A, closed circles, 1.0 ± 6%, n = 6 p = 0.01, Bonferroni correction). In contrast, the GluN2A selective concentration of NVP-AMM077 (NVP, 0.1 μM) was insufficient for blockade of LTD (Fig. 1B, open triangles, 24 ± 3%), which was indistinguishable from that in the control (n = 6, p = 1, Bonferroni correction). Similarly, the GluN2D-preferring compound UBP145 (10 μM, Fig. 1C) had no effect on LTD (18 ± 4%, n = 6, p = 1, Bonferroni correction).

### 3.2 Inhibition of GluN2B receptors is sufficient for blockage of LTD

**Fig. 2:**
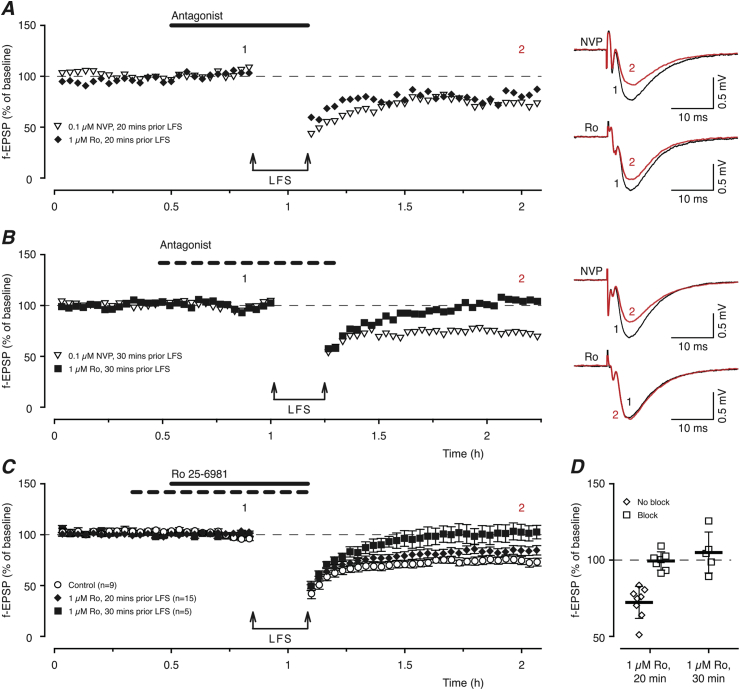
Blockade of GluN2B-containing NMDARs is sufficient to prevent induction of LTD. (**A**) A 20 min application of 1 μM Ro (filled diamonds) or 0.1 μM NVP (open triangles) prior to LFS is insufficient to block LTD. (**B**) A 30 min pre-application of 1 μM Ro (filled squares) inhibits LTD whereas a similarly long application of 0.1 μM NVP (open triangles) has no effect on LTD. (**C**) Summary of the experiments using 1 μM Ro showing that a 30 min pre-application time is necessary for complete blockade of LTD (filled squares, n = 5) whereas a 20 min pre-application is insufficient (filled diamonds, n = 15). (**D**) The data show the 4 min estimates of LTD (1 h post LFS) from single experiments. Robust inhibition of LTD is only seen with longer pre-incubation with Ro whereas a shorter application time leads to variable effects.

The results of experiments using GluN2A- and GluN2D-preferring antagonists suggested that these subunits are not required for the induction of LTD. We therefore tested whether Ro 25-6981 (Ro, Fig. 2), which effectively blocks GluN2B-containing diheteromers selectively at a concentration of 1 μM, is able to block LTD. A 20 min pre-application of Ro had a variable effect on LTD; in 8 experiments it had no effect whereas in 7 experiments a significant block was observed (Fig. 2D). In contrast, when Ro was pre-applied for 30 min, complete inhibition of LTD was observed in all experiments. Representative single example experiments for Ro (filled symbols) and NVP (open symbols) are shown for a 20 min pre-incubation (Fig. 2A) and a 30 min pre-incubation (Fig. 2B). This clearly shows the time-dependence of the block of LTD by Ro but not by NVP. The pooled data for Ro experiments, showing no overall significant effect with 20 min pre-incubation (15 ± 4% vs 26 ± 4%, n = 15, p = 0.3, Bonferroni correction) but complete inhibition with 30 min pre-incubation (2 ± 4%, n = 5, p = 0.03, Bonferroni correction) when compared to control experiments (26 ± 4%) are presented in Fig. 2C, D.

### 3.3 Effects of NMDAR antagonists on the induction of STP and LTP

**Fig. 3:**
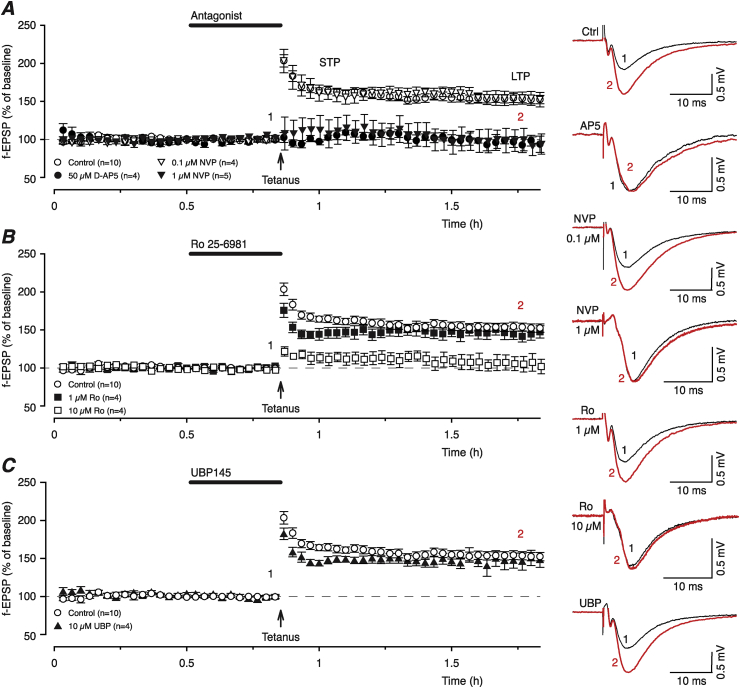
GluN2B- and GluN2D-containing receptors are involved in the induction of STP. (**A**) Tetanic stimulation (100 Hz, 1 s) induced both STP and LTP (open circles, n = 10). 50 μM D-AP5 (filled circles, n = 4) reliably blocks the induction of STP and LTP. 0.1 μM NVP (open triangles, n = 4) has no effect on the induction of potentiation whereas 1 μM NVP blocks both STP and LTP (filled triangles, n = 5). (**B**) STP is significantly reduced after pre-incubation with 1 μM Ro (filled squares, n = 4) whereas LTP is not affected. 10 μM Ro (open squares, n = 4) completely abolishes LTP. (**D**) 10 μM UBP (filled triangles, n = 4) reduces STP but spares LTP.

In control experiments, tetanisation (100 Hz, 1 s, HFS) induced STP that declined with a τ value of 5.2 min (CI 3.7–8.5 min) to a stable level of LTP (Fig. 3A, open circles, LTP = 53 ± 5%, n = 10, p < 0.05 when compared to the pre-HFS baseline, paired *t*-test). Both STP and LTP were abolished by pre-application of 50 μM AP5 (Fig. 3A, filled circles, n = 4). By the end of experiments using AP5, potentiation was 2 ± 8% and significantly different from the control LTP (p < 0.001, Bonferroni correction).

In contrast to AP5, a low concentration of NVP (0.1 μM) had no effect on the induction of STP or LTP (open triangles, Fig. 3A) and STP declined with a τ value of 3.3 min (CI 1.9–11.1 min, p = 0.32 vs control) to a LTP level of 54 ± 9% (n = 4, p = 1 vs control, Bonferroni correction). However, both STP and LTP were completely blocked by a high concentration of NVP (1 μM, n = 5, filled triangles, Fig. 3A, LTP = −0.6 ± 13%).

Ro had concentration-dependent effects on synaptic potentiation. Thus, 1 μM Ro reduced the decay time of STP significantly (filled squares, Fig. 3B, τ = 1.2, CI 1.14–1.26 min, p = 0.03 vs control) without affecting induction of LTP (45 ± 8%, n = 4, p = 1 vs control, Bonferroni correction). However, LTP was blocked completely by 10 μM Ro (open squares, Fig. 3B, n = 4, LTP = 2 ± 10%). Similarly to the low concentration of Ro, UBP145 reduced the decay time of STP significantly (τ = 1.6, CI 0.98–15.5 min, p = 0.04 vs control) without affecting LTP (Fig. 3C filled triangles, LTP = 47 ± 7%, p = 1 vs control, Bonferroni correction).

## 4 Discussion

The present study investigated the role of NMDAR subunits in LTD and LTP in P14 animals using GluN2A, 2B and 2D subunit-preferring antagonists. NMDARs are most commonly composed of two GluN1 and two GluN2 subunits and it is the identity of the GluN2 subunit, which contains the glutamate binding site, that confers the receptors with distinct biophysical properties, determining their affinity to binding glutamate, regulating the probability of channel opening and the decay kinetics of macroscopic currents and distinct pharmacological properties (Erreger et al., 2005, Monyer et al., 1994, Monyer et al., 1992, Vicini et al., 1998). GluN1 subunits bind the co-agonists glycine or d-serine and their activation is obligatory to the channel function. The voltage sensitivity of the channel is due to the Mg^2+^ block that gets relieved during depolarizing membrane potentials (Ault et al., 1980, MacDonald and Wojtowicz, 1980, Mayer et al., 1984, Nowak et al., 1984). The sensitivity of channels to the Mg^2+^ block is also dependent on the identity of GluN2 subunits, which can be found in a functional receptor in either “homomeric” (i.e. both GluN2 subunits are identical) or in “heteromeric” (i.e. two, different GluN2 subunits are found in the receptor assembly) form. Thus, NMDARs, containing two identical GluN2 subunits (e.g. 2GluN1/2GluN2A) are frequently referred to as diheteromeric whereas receptors that include different GluN2 subunits (e.g. 2GluN1/GluN2A/GluN2B) are referred to as triheteromeric (Hansen et al., 2014, Paoletti and Neyton, 2007). The expression of NMDARs is regulated both regionally in the brain and throughout development and maturation of an animal (Buller et al., 1994, Monyer et al., 1994, Thompson et al., 2002, Watanabe et al., 1992). Thus, expression of GluN2As starts postnatally and then increases with development to steady adult levels in the hippocampus. In contrast, GluN2B subunits are expressed highly across the different developmental stages whereas a low expression of the GluN2Ds has been observed postnatally. GluN2C subunits are not expressed in rodent hippocampus, postnatally.

### 4.1 GluN2 subunit-preferring antagonists

In the present study, in addition to AP5, which is routinely used to block synaptic plasticity at the Schaffer collateral – CA1 synapse (Collingridge et al., 1983, Dudek and Bear, 1992), we used three subunit-preferring antagonists: NVP-AAM077 (Auberson et al., 2002), Ro 25-6981 (Fischer et al., 1997) and UBP145 (Costa et al., 2009, Irvine et al., 2012) to block GluN2A-, GluN2B- and GluN2D-containing receptors, respectively. We have previously characterized NVP, Ro and UBP in detail and used these antagonists to determine subunit composition of NMDARs involved in the induction of STP and LTP in adult hippocampus (Volianskis et al., 2013a). NVP and UBP145 bind to the glutamate-binding site of NMDARs with differential potency at the receptors dependent on the identity of the GluN2 subunit, whereas Ro is a negative allosteric modulator of GluN2B-containing NMDARs with a complex mode of action (Karakas et al., 2011).

NVP is about 10-fold more potent at the GluN2A-containing NMDARs when compared to the GluN2B and can, at a concentration of 0.1 μM, discriminate between these receptor subtypes as shown previously in recombinant receptor assays (Feng et al., 2004, Frizelle et al., 2006, Volianskis et al., 2013a). NVP blocks GluN2D-containing receptors also, where it presents with intermediate potency (GluN2A > GluN2D > GluN2B, rank order potency). UBP145 is ∼10-fold more potent at the GluN2D subunits than at the other receptor subtypes (GluN2D > GluN2A = GluN2B) and at a concentration of 10 μM it blocks ∼90% of recombinant GluN2D-containing receptors expressed in HEK293 cells (Volianskis et al., 2013a). Ro is the most selective of the three subunit-preferring antagonists that were used in this study. At concentrations of up to 1 μM it blocks diheteromeric GluN2B-containing receptors, with an IC_50_ value in the low nanomolar range, although its potency is inversely dependent on the concentration of the agonist (Volianskis et al., 2013a). Furthermore, at low agonist concentrations, Ro can potentiate diheteromeric GluN2B-containing receptor response, a feature that is shared with ifenprodil and not seen at the other receptor subtypes (Fischer et al., 1997, Hansen et al., 2014, Volianskis et al., 2013a). At concentrations of 3–30 μM, Ro blocks triheteromeric NMDARs containing both GluN2A and GluN2B subunits, whereas at higher concentrations (>30 μM) it starts showing inhibitory effects at the GluN2A-containing diheteromers (Fischer et al., 1997, Hansen et al., 2014, Volianskis et al., 2013a). At concentrations of up to 30 μM Ro does not inhibit GluN2D subunits (higher concentrations of this antagonist have not been tested at the GluN2D subunit).

In summary, although NVP, Ro and UBP145 have limited selectivity towards the different NMDA receptor subtypes, a direct comparison of the actions of these antagonists at appropriate concentrations enables firm conclusions to be drawn about the involvement of these receptor subtypes in the induction of synaptic plasticity.

### 4.2 NMDARs in synaptic plasticity

Although there is no doubt about the involvement of NMDARs in generating synaptic plasticity in the CA1 area of the hippocampus (Bliss and Collingridge, 2013, Bliss and Collingridge, 1993, Collingridge et al., 1983, Volianskis et al., 2015), considerable disparity remains in allocating selective functional roles for the specific NMDAR-subunits in the induction of synaptic plasticity. Some of the differences in the results might be explained by differences in experimental conditions, animal species and their developmental stage (Bartlett et al., 2007, Bartlett et al., 2011, Berberich et al., 2007, Berberich et al., 2005, Hendricson et al., 2002, Köhr et al., 2003, Liu et al., 2004, Massey et al., 2004, Morishita et al., 2007). In addition, as mentioned previously, allosteric modulators such as ifenprodil and Ro can function as potentiators at low glutamate concentrations (Fischer et al., 1997, Hansen et al., 2014, Volianskis et al., 2013a), potentially confusing the results. Furthermore, various induction paradigms may engage NMDARs subtypes differently due to their distinct biophysical properties and localization.

In the current study we have used two of the most-common induction paradigms, i.e. low frequency stimulation (1 Hz for 15 min) and high frequency tetanisation (100 Hz for 1 s) to induce LTD and STP/LTP respectively.

### 4.3 NMDAR subunits in LTD

The role for GluN2B receptors in the induction of LTD was originally suggested by the observation that both Ro and ifenprodil can block LFS-induced LTD in slices from adult rat perirhinal cortex (Bartlett et al., 2007, Massey et al., 2004) and from 3 to 4 week old rat hippocampus (Liu et al., 2004). However, in other experiments, Ro and ifenprodil were unable to block LTD in rat hippocampal slices from 3 to 4 week old animals (Morishita et al., 2007), for reasons that are still unclear.

In the present study, we focused on LTD at a slightly earlier developmental stage, 2 weeks of age. Our observation that Ro completely blocked LTD at a concentration selective for GluN2B-containing diheteromers is consistent with the canonical view that this NMDAR-subtype can mediate LTD induction. However, blockade of this subtype is not invariably sufficient to inhibit LTD at this developmental stage (Bartlett et al., 2007) with other factors such as slice orientation playing a role (Bartlett et al., 2011).

We conclude therefore, that GluN2B receptors are required for LTD under some circumstances but their involvement may be compensated for under other conditions. Developmental changes in the expression of GluN2B receptors may be one determinant but other factors, such as the level of cholinergic modulation (Bartlett et al., 2011), may be involved. In the present study, we observed no effect on LTD with concentrations of NVP and UBP145 that are selective for GluN2A and GluN2D, respectively. This supports the idea, that GluN2B can be the major determinant of LTD.

### 4.4 NMDAR subunits in LTP

The role of NMDAR subunits in the induction of LTP is also highly controversial (e.g. Bartlett et al., 2007, Berberich et al., 2007, Berberich et al., 2005, Li et al., 2007, Liu et al., 2004, Massey et al., 2004, Volianskis et al., 2013a). In the current study, LTP was completely blocked by either 50 μM AP5,1 μM NVP or 10 μM Ro. However, 1 μM Ro and UBP145 were ineffective. These data suggest that triheteromeric NMDARs, containing both GluN2A and GluN2B subunits, play an important role in the induction of LTP at this stage of development, as previously shown in adults (Volianskis et al., 2013a).

### 4.5 NMDAR subunits STP

STP, the transient enhancement in synaptic transmission that overlaps with LTP, has been shown to have a different NMDAR-dependence compared to LTP in adult rats (Volianskis et al., 2013a). More specifically, in slices prepared from adult rats, STP comprises two overlapping components; a fast component, termed STP_1_, that has the same sensitivity to antagonists as LTP, and a slow component, termed STP_2_, that is sensitive to both Ro and UBP145. It was therefore proposed that STP_2_ involves both GluN2B and GluN2D subunits. A similar slow component of STP with high sensitivity to both Ro and UBP was observed in the present study. Therefore STP_2_ shows no obvious developmental regulation. Its function remains to be determined although a role in working memory has been postulated (Volianskis et al., 2013a, Volianskis et al., 2013b). In terms of STP_1_ the parallel developmental regulation in both its sensitivity and that of LTP to NVP reinforces the view that these two forms of synaptic plasticity are closely associated with one another.

### 4.6 Concluding remarks

In this study on slices obtained from P14 hippocampus, using GluN2A, 2B and 2D subunit-preferring concentrations of NVP, Ro and UBP145, we have demonstrated that activation of GluN2B-containing receptors can be sufficient for the induction of LTD. We have also shown that GluN2B- and GluN2D-containing receptors are involved in the induction of a component of STP. Finally, we have presented evidence that GluN2A/2B triheteromers are the dominant form involved in LTP. These data support the view that different NMDA receptor subtypes play distinct roles in various forms of synaptic plasticity. They also demonstrate that a single subunit, in this case GluN2B, is involved in multiple forms of synaptic plasticity at the same set of synapses.

## Funding

This study was supported by the MRC (G0601812), the BBSRC (BB/L001977/1) and the NIH (R01MH060252).
